# Colonisation potential of microplastic particles containing organic pollutants by a river-isolated environmental *Acinetobacter baumannii*

**DOI:** 10.2478/aiht-2026-77-4049

**Published:** 2026-06-30

**Authors:** Svjetlana Dekić Rozman, Maja Vujić, Jasna Hrenović, Vesna Gvoić, Miljana Prica, Aleksandra Tubić

**Affiliations:** University of Helsinki Faculty of Agriculture and Forestry, Department of Microbiology, Helsinki, Finland; University of Zagreb Faculty of Science, Department of Biology, Zagreb, Croatia; University of Novi Sad Faculty of Sciences, Department of Chemistry, Biochemistry, and Environmental Protection, Novi Sad, Serbia; University of Novi Sad Faculty of Technical Sciences, Department of Graphic Engineering and Design, Novi Sad, Serbia

**Keywords:** bacteria, benzene derivatives, microplastics, polyethylene, water, bakterija, derivati benzena, mikroplastika, polietilen, voda

## Abstract

Microplastics in aquatic environments raise concern about their role as potential carriers of pathogens and organic pollutants. This study investigates the survival of the extensively drug-resistant *Acinetobacter baumannii* in the presence of selected priority substances including benzene derivatives (trichlorobenzene, pentachlorobenzene, and hexachlorobenzene), trifluralin, and primary polyethylene microplastics. The results indicate that the amount of adsorbed priority substances on microplastics from a mixture solution is slightly lower than the adsorption from individual solutions. Furthermore, GC/MS analysis shows that other chemicals used in plastic manufacturing can be released from microplastics into the environment over time and should be taken into account when assessing the environmental impact of microplastics. The proliferation of the environmental Sava 4 *A. baumannii* strain was not affected by the presence of benzene derivatives and microplastic particles at concentrations of up to 10 g/L in the water medium, and microscopy confirmed that it can colonise and form a biofilm on microplastic particles with adsorbed benzene derivatives, which demonstrates that microplastics have the potential to spread pollutants and potentially harmful bacteria over long distances and introduce them into various aquatic environments.

## Abstract (bs)

Mikroplastika, kao sve prisutnije zagađivalo u vodenim ekosustavima, izaziva zabrinutost zbog svoje potencijalne uloge vektora patogena i organskih zagađivala. U ovoj se studiji istraživalo preživljavanje višestruko rezistentnoga bakterijskog soja *Acinetobacter baumannii* Sava 4 u prisutnosti prioritetnih tvari, uključujući derivate benzena (triklorobenzeni, pentaklorobenzen i heksaklorobenzen), kao i trifluralin, te polietilen u obliku mikroplastike. Rezultati su pokazali da je količina adsorbiranih prioritetnih tvari na mikroplastici, kada su aplicirane u obliku smjese, nešto manja u odnosu na sorpciju iz pojedinačnih otopina. Nadalje, rezultati GC/MS analize neciljanih spojeva upućuju na to da se kemikalije iz proizvodnje plastike ili iz plastičnih proizvoda mogu postupno otpuštati u okoliš, zbog čega bi se taj čimbenik svakako morao uzeti u obzir pri procjeni ekološkog utjecaja mikroplastike. Prisutnost benzenskih derivata i čestica mikroplastike u koncentracijama do 10 g/L nije značajno utjecala na preživljavanje bakterije *A. baumannii* Sava 4 u vodenom mediju. Mikroskopska analiza potvrdila je sposobnost *A. baumannii* Sava 4 da kolonizira čestice mikroplastike na koje su adsorbirani derivati benzena te da na njima formira biofilm. Ovi nalazi potvrđuju da mikroplastika može poslužiti kao medij za širenje onečišćujućih tvari i potencijalno patogenih bakterija na velike udaljenosti te za njihovo uvođenje u različite vodene ekosustave.

Microplastics are plastic particles smaller than 5 mm in diameter that were identified in the 2014 United Nations Environment Programme (UNEP) Yearbook as one of the ten emerging pollutants that potentially threaten human health and other organisms (1, 2). Commercial plastics usually consist of synthetic polymers, most often polyethylene (PE), which resist degradation (3,4,5) and greatly burden the environment (6). With an average diameter of 125–355 µm, these particles can easily escape conventional wastewater treatment plant (WWTP) filters and enter water systems (7). Numerous studies have already confirmed the harmful effects of microplastics on marine and terrestrial ecosystems (3, 8).

To make things worse, more than 200 types of organic compounds have been identified on microplastics, most of which are persistent organic pollutants (9). These include pharmaceuticals (10,11,12), bisphenol (13), 17β-oestradiol (14), benzene, toluene, ethyl benzene, and xylene (BTEX) (15), pesticides (16, 17), polycyclic aromatic hydrocarbons (18,19,20), organophosphates (21), phthalates (22), and phenols (23, 24). Because of the ability to adsorb other pollutants, microplastics are now also considered potential vectors for pathogenic and antibiotic-resistant bacteria in natural waters (3, 9, 25, 26).

Study results vary, with some types of microplastic being even more toxic in combination with other pollutants, while other studies report no additional synergistic effect (3, 27,28,29,30).

Given these contradictory reports and the risk that microplastics should behave as a substrate for pathogenic organisms (3, 25, 26, 31, 32), we wanted to test the hypothesis that polyethylene microparticles could serve as a vector for the transport of organic pollutants and harmful bacteria and introduce them into various aquatic environments by investigating whether the presence of organic pollutants influences bacterial ability to colonise microplastics.

## MATERIALS AND METHODS

We therefore studied the survival of the extensively drug-resistant *Acinetobacter baumannii* Sava 4 strain on primary polyethylene (PE) microplastic in the presence of six priority substances according to the EU Directive 2013/39/EU, namely trichlorobenzenes (1,2,3-TeCB; 1,3,5-TeCB; 1,2,4-TeCB), pentachlorobenzene (PeCB), hexachlorobenzene (HeCB), and trifluralin (TFL) (33).

The *A. baumannii* Sava 4 was previously isolated from the Sava River downstream of the wastewater treatment plant in Zagreb, Croatia (34). This isolate is characterised by extensive carbapenem resistance and is genetically related to clinical isolates belonging to the sequence type 195 of the international clone 2. *A. baumannii* has come into the focus of our study because it bears a growing threat to health care (35, 36) as one of the priority environmental pathogens capable of forming biofilm on plastic surfaces (34, 37,38,39,40,41,42,43,44,45,46,47,48).

### Chemicals

For the purposes of this study, we used standard PE microparticle powder (PEp) from Thermo Fischer Scientific (Basel, Switzerland; Cat. No. A10239.36; CAS No. 9002-88-4) and PE microbeads isolated from two personal care products (designated as PE_PCP_1 and PE_PCP_2) by mixing each product with boiled water, followed by separation based on density differences using NaCl, and removal of organic components with 30 % H_2_O_2_ at room temperature for 24 h. The samples were then diluted with distilled water, filtered through a membrane filter (0.45 µm), placed in a clean Petri dish, and dried at room temperature.

Previous studies using similar protocols (2, 49, 50) have demonstrated that these treatments do not alter the polymer backbone of polyethylene, which was confirmed in this study by Fourier-transform infrared (FTIR) spectroscopy. In addition to FTIR, the selected microplastics were further characterised with scanning electron microscopy (SEM) and Brunner-Emmett-Teller (BET) surface area analysis, described in detail in our previous work (51). All three microplastic adsorbents were composed of the same mesoporous polymer type but differed in size and structure. Thermo Fisher PEp size ranged from 49.7 to 259 μm in diameter, PE_PCP_1 (80–185 µm) had the lowest specific area and mesopore volume, and PE_PCP_2 particles were much larger than those of the other two adsorbents (244–358 µm), indicating the formation of agglomerates. Agglomeration may reduce the accessible surface area and limit the availability of adsorption sites.

The benzene derivatives used in this study were all high-purity Pestanal^®^ products obtained from Sigma-Aldrich (Burlington, MA, USA). These benzene derivatives were selected based on their different octanol/water partition coefficients and water solubility. Stock solutions (1 mg/mL) of each compound were prepared in methanol.

The organic solvents used in this study for organic residue analysis were J.T. Baker (Avantor, Center Valley, PA, USA), while hexane and methanol were purchased from Avantor Performance Materials B.V., Deventer, The Netherlands. Analytical grade hydrogen peroxide was obtained from Sigma-Aldrich.

### Adsorption experiments

Adsorption of chemicals onto microplastic particles was determined in batch experiments performed with distilled water (pH 7.0±0.1) in 40 mL glass vials at room temperature (25 °C). Seven aliquots of 30 mL distilled water were each mixed with one of the tested compounds (1,2,3-TeCB, 1,2,4-TeCB, 1,3,5-TeCB, PeCB, HeCB, TFL) and their mixture to achieve an initial concentration of 100 µg/L. Due to the low water solubility of the investigated benzene derivatives, stock solutions were prepared in methanol and subsequently diluted with distilled water to obtain homogeneous aqueous solutions at environmentally relevant concentrations. Ten milligrams of selected microplastics were added to each solution. The vials were capped and shaken with an IKA^®^ Orbital Shaker KS 501 Digital (IKA-Werke GmbH & Co. KG, Staufen im Breisgau, Germany) at 150 rpm for 48 h, based on our previous findings (51) indicating maximum adsorption at this rate. After shaking, the samples were filtered through a 0.45 µm membrane filter to separate the microplastics from the liquid phase. The retained particles were air-dried (at 22–24 °C) for 24 h and stored in 2 mL glass vials for microbiological studies. Filtered water samples were prepared for gas chromatography with an electron capture detector (GC/µECD) to determine the residual concentrations of compounds in the aqueous phase.

Adsorption was quantified indirectly by calculating the difference between initial and remaining concentrations. The amount of adsorbed compounds is expressed as adsorption capacity at equilibrium (q_e_, µg/g), representing the mass of adsorbate bound to microplastic particles per unit mass of adsorbent.

### Microbiological analysis

The *A. baumannii* Sava 4 isolate was grown overnight on TTCTergitol agar (Biolife Italiana S.r.l., Milan, Italy) at 42 °C. At the beginning of the experiment, the bacterial count was 7.0±0.1 log CFU/mL. Its biomass was transferred using a full 10-µL loop and suspended in 10 mL of sterile physiological solution (0.85 % NaCl). Two millilitres of the bacterial suspension were transferred to 300 mL of Mueller-Hinton broth (Biolife) as a standard nutrient medium that supports reproducible bacterial growth and allows assessment of survival and biofilm formation under controlled conditions. Sterile PlateOne^®^ Deep 96-well 2 mL polypropylene plates (USA Scientific, Inc., Ocala, FL, USA) were used to determine the minimum inhibitory concentration of the pesticides adsorbed on the microplastics. Microplastic particles (10 mg) were suspended in 1 mL of bacterial suspension to obtain the highest microplastic concentration (10 g/L) and added to the first well. All other wells were filled with 0.5 mL of the bacterial suspension. The first well was stirred and 0.5 mL added to the next well, reducing the microplastic concentration by half. The same procedure was repeated until the lowest concentration was reached. The concentrations tested were 10,000, 5,000, 2,500, 1,250, 625, 312, 156, 78, 39, 20, and 10 mg of microplastic particles per litre (mg/L).

The microtitre plates were incubated in the dark at 22 °C for five days. Bacterial suspension without microplastics was considered positive control because preliminary experiments showed no observable effect of benzene-free polyethylene microparticles on the survival of *A. baumannii* Sava 4. Bacterial counts were determined in the positive control and in all wells containing microplastics after one and five days of exposure.

A subsample of 100 µL was transferred to 900 µL of physiological solution, further diluted (up to 10^−8^), inoculated onto selective TTC-Tergitol agar (Biolife), and incubated at 42 °C for 24 h. The experiments were performed in technical triplicate. Bacterial abundance was log_10_ transformed and expressed as log CFU/mL.

### Desorption experiments

Following the microbiological analysis, desorption experiments with microplastics were conducted to determine possible leaching of adsorbed benzene derivatives and other plastic additives upon contact with microorganisms. All experiments were carried out in 40 mL glass vials shaken at 150 rpm at room temperature. Ten milligrams of microplastic (previously loaded with a mixture of the tested compounds) were mixed with 30 mL of Mueller-Hinton broth prepared under identical conditions as in the microbiological assays but without bacterial inoculation. Desorption was monitored for one and five days to assess the potential release of organic pollutants into the medium during the same exposure period used in the microbiological experiments. The filtered samples were prepared for quantitative and qualitative gas chromatographic analysis. The surface of the dried microplastic (PEp, PE_PCP_1, and PE_PCP_2) was examined with a TM3030 scanning electron microscope (SEM) (Hitachi High-Tech Corporation, Tokyo, Japan) to assess potential surface changes and the presence of residual microbial structures after the desorption.

### Microscopy

In this study, bacterial viability was evaluated using cultivation-based methods, while microscopy served to complement morphological observations of cell-particle interactions. Bacterial suspensions with microplastics (10 µL) were added to slides, dried, and fixed with a brief flame treatment. The fixed slides were stained with carbol-fuchsin dye (Biognost, Zagreb, Croatia) for 10 sec. For microscopy we used an Olympus CX2 (Olympus Corporation, Tokyo, Japan) and photographed with a Canon EOS 400D (Canon Inc., Tokyo, Japan) at 1000× magnification.

SEM analysis was used to qualitatively assess bacterial attachment and spatial distribution on microplastic surfaces after sample preparation, which included washing steps to remove non-attached cells. It should be noted that SEM does not differentiate between live and dead cells and therefore does not provide information on cell viability. Microplastic particles for SEM were fixed in 2.5 % glutaraldehyde in phosphate-buffered saline and prepared according to the standard procedure. After removal of the fixative, the samples were rinsed in phosphate buffer, fixed in 1 % osmium tetroxide in distilled water, rinsed again in phosphate buffer, and then dehydrated sequentially with ethanol to absolute ethanol. The samples were then dried with hexamethyldisilazane, coated with carbon, and analysed under the microscope.

### Gas chromatography

The adsorbed benzene derivatives were quantified with the Agilent^®^ 6890 gas chromatograph coupled with electron capture detector (GC/µECD) (Agilent Technologies, Inc., Wilmington, DE, USA) after liquid-liquid extraction with hexane to determine benzene derivative concentrations remaining in the solution, following the procedures described by the United States Environmental Protection Agency (US EPA) (52,53,54). Briefly, a 0.5 mL aliquot of the extract was transferred to a GC vial and spiked with 1 µL of pentachloronitrobenzene (PCNB) solution (Sigma-Aldrich; Cat. No. 40156) as an internal standard (final concentration of 25 ng/mL). The GC oven temperature programme started at 70 °C, held for 1 min, and was then ramped at 20 °C/min to 180 °C. The temperature was then increased at 10 °C/min to 230 °C and maintained for 3 min. Finally, the oven was ramped at 5 °C/min to 300 °C and held for 5 min. Two microlitres of the sample extract were injected in splitless mode. The injector temperature was 250 °C, and the detector temperature 300 °C. The quality control parameters are listed in Table 1. Samples from microbiological assays were not subjected to quantitative GC analysis, as this study focused on adsorption/desorption behaviour and its indirect influence on bacterial survival.

**Table 1:** Quality parameters data for GC/µECD analysis of benzene derivatives

**Benzene derivative**	**MDL (ng/L)**	**PQL (ng/L)**	**Accuracy (%)**	**Precision (RSD %)**
1,2,3-TeCB	3.72	6.41	98.82	8.81
1,2,4-TeCB	3.33	5.84	102.2	7.15
1,3,5-TeCB	3.02	5.36	104.1	7.22
PeCB	3.02	7.62	108.0	12.7
HeCB	2.18	5.45	97.70	11.9
TFL	3.87	9.67	100.0	11.7

1,2,3-TeCB – 1,2,3-trichlorobenzene; 1,2,4-TeCB – 1,2,4-trichlorobenzene; 1,3,5-TeCB – 1,3,5-trichlorobenzene; HeCB – hexachlorobenzene; MDL – method detection limit; PeCB – pentachlorobenzene; PQL – practical quantitation limit; TFL – trifluralin

After liquid-liquid extraction with hexane, 0.5 mL of the extract was transferred to a vial for qualitative analysis with a gas chromatograph - mass spectrometer (GC-MS; Agilent Technologies 7890A GC/5975C VL MSD system) to identify non-target compounds released from microplastics. The chromatographic conditions were as follows: the initial oven temperature of 70 °C was held for 2 min and then ramped at 25 °C/min to 150 °C, immediately followed by ramping at 3 °C/min to 200 °C and at 8 °C/min to 280 °C, where it was held for 10 min. Two microlitres of the sample extract were injected in splitless mode. The injector temperature was 250 °C, and the detector temperature 300 °C.

### Statistical analysis

All experiments were performed in technical triplicate. Bacterial abundance data were log_10_ transformed before statistical analysis, which was run on Statistica 13.3 (TIBCO Software, Inc., Palo Alto, CA, USA). Pairwise comparisons were done using factorial analysis of variance (ANOVA), followed by Duncan's test. Statistical significance was set at p<0.05.

## RESULTS AND DISCUSSION

### Abiotic interactions between polyethylene microplastics and benzene derivatives

**Figure 1:**
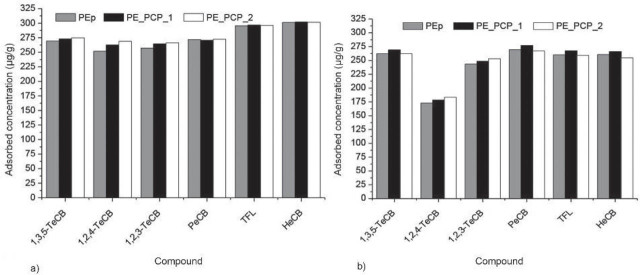
Mean (±SD) adsorbed benzene derivative per unit mass of adsorbent (µg/g) after 48 h of microplastics exposure: a) in individual solutions and b) in the mixture (n=3). 1,2,3-TeCB – 1,2,3-trichlorobenzene; 1,2,4-TeCB – 1,2,4-trichlorobenzene; 1,3,5-TeCB – 1,3,5-trichlorobenzene; HeCB – hexachlorobenzene; PE_PCP_1 – PE microparticles from the personal care product 1; PE_PCP_2 – PE microparticles from the personal care product 2; PeCB – pentachlorobenzene; PEp – standard PE microparticles (Thermo Fisher); TFL – trifluralin

Figure 1 shows higher benzene derivative adsorption on microparticles from single (Figure 1a) than the mixed solution (Figure 1b), ranging from 252 to 302 µg/g vs. 173 to 277 µg/g, respectively. This difference can be attributed to the competition between the individual compounds in the mixture, as reported elsewhere (55,56,57).

The adsorption affinities for PE microparticles isolated from personal care products are slightly higher than those for the standard PE, suggesting that processing during production may have altered their properties, which has also been noted by other authors (19, 50, 51, 58,59,60).

**Figure 2:**
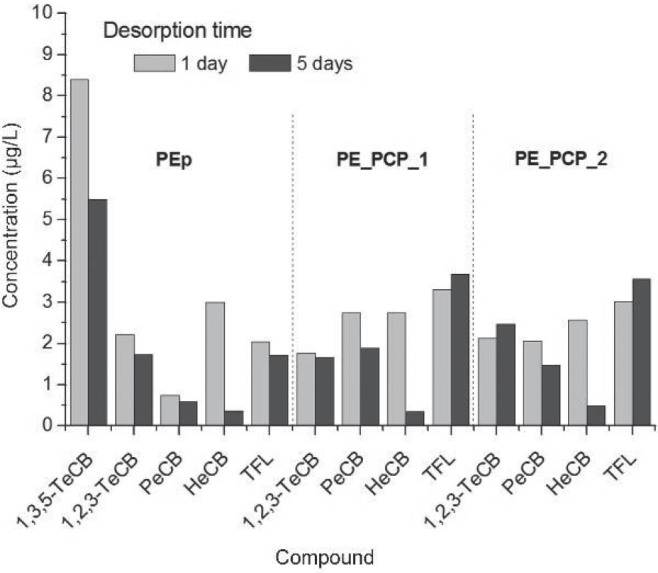
Mean (±SD) desorbed concentrations of benzene derivatives in aqueous medium (µg/L) after 1 and 5 days of desorption. 1,2,3-TeCB – 1,2,3-trichlorobenzene; 1,2,4-TeCB – 1,2,4-trichlorobenzene; 1,3,5-TeCB – 1,3,5-trichlorobenzene; HeCB – hexachlorobenzene; PE_PCP_1 – PE microparticles from the personal care product 1; PE_PCP_2 – PE microparticles from the personal care product 2; PeCB – pentachlorobenzene; PEp – standard PE microparticles (Thermo Fisher); TFL – trifluralin

Figure 2 shows the desorption of benzene derivatives from PEp, PE_PCP_1, and PE_PCP_2 after one and five days. The desorption of chlorinated benzenes was very low, and that of 1,2,4-TeCB below the detection limit. This may be attributed to its hydrophobicity, strong affinity for polyethylene, relatively low solubility in water, and partial transformation or degradation. Similar behaviour of hydrophobic organic contaminants in aqueous systems has already been associated with a combination of adsorption potential, transformation, and analytical limitations (18, 51, 56, 61). 1,3,5-TeCB was desorbed only from the standard PE microparticles (PEp), with concentrations ranging from 5.5 to 8.4 µg/L. 1,2,3-TeCB, PeCB, HeCB, and TFL were released from all three PE microparticles but at very low concentrations (up to 3.6 µg/L).

In general, higher concentrations of desorbed benzene derivatives were measured on day 1 than day 5, which is in line with the desorption dynamics reported elsewhere (11, 62). Overall, our findings suggest that microplastics act as relatively stable reservoirs of hydrophobic pollutants, with limited short-term release into the aqueous phase.

The non-target substances detected in the culture medium after desorption can be classified as industrial chemicals used in the pharmaceutical or cosmetic industries (e.g. benzaldehyde and acetophenone), plasticisers [e.g. dibutyl phthalate and bis(2-ethylhexyl) phthalate], or solvents such as benzyl alcohol (Table 2). These non-target compounds may affect the adsorption of benzene derivatives through competitive interactions at the microplastic surface, although this aspect was beyond the scope of this study. However, their presence should be considered when assessing the environmental impact of microplastics, as outlined in the recent comprehensive report by Fred-Ahmadu et al. (61).

**Table 2:** Presence of non-target compounds in culture medium after desorption of 1 and 5 days

**Compound**	**Microplastic**					
	**PEp after 1 d**	**PEp after 5 d**	**PE_PCP_1 after 1 d**	**PE_PCP_1 after 5 d**	**PE_PCP_2 after 1 d**	**PE_PCP_2 after 5 d**
Benzaldehyde	+	+	+	+	+	+
2-ethyl hexanol	+	+	+	+	+	+
Benzyl alcohol	+	+				+
Phenylethyl alcohol	+	+				
1-Dodecanol	+	+				
Prophylbenzene	+	+				
Acetophenone	+	+	+	+	+	+
Diethyl phthalate	+	+	+	+	+	+
Dibutyl phthalate	+	+	+	+		
Bis (2-ethylhexyl) phthalate						+
1,2-Benzenedicarboxylic acid		+				+

“+” indicates detection of the compound. PEp – standard PE microparticles (Thermo Fisher); PE_PCP_1 – PE microparticles from the personal care product 1; PE_PCP_2 – PE microparticles from the personal care product 2

### Microbiological findings

**Figure 3:**
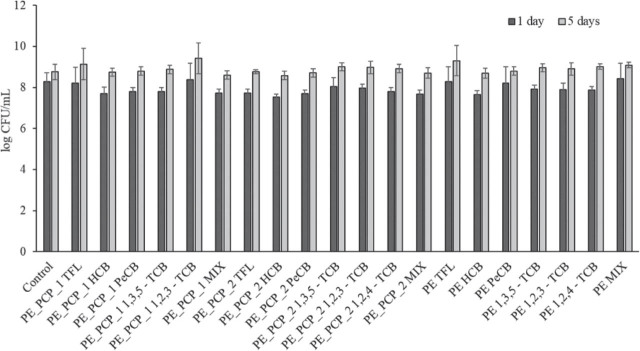
*A. baumannii* Sava 4 counts after the short- (one day) and long-term (five days) exposure to the highest concentration of microplastics (10 g/L) containing TFL; HCB; PeCB; 1,2,3-TCB; 1,2,4-TCB; 1,3,5-TCB separately and in a mixture (n=3, mean value ± SD). The starting *A. baumannii* Sava 4 abundance was 7.0±0.1 log CFU/mL. 1,2,3-TeCB – 1,2,3-trichlorobenzene; 1,2,4-TeCB – 1,2,4-trichlorobenzene; 1, 3, 5 - T e C B – 1,3,5-trichlorobenzene; HeCB – hexachlorobenzene; PE_PCP_1 – PE microparticles from the personal care product 1; PE_PCP_2 – PE microparticles from the personal care product 2; PeCB – pentachlorobenzene; PEp – standard PE microparticles (Thermo Fisher); TFL – trifluralin

Figure 3 shows that *A. baumannii* Sava 4 counts after one and five days of incubation with PE microparticles coated with benzene derivatives did not significantly differ from positive control, regardless of the type of microplastic or benzene derivative. In other words, microplastic particles coated with pesticides (benzene derivatives) had no inhibitory effect on *A. baumannii* Sava 4, nor did the free benzene derivatives or other non-target chemicals leach from microparticles. Although some bacterial genera are known to tolerate or degrade chlorinated aromatic compounds, information on the susceptibility of extensively drug-resistant *A. baumannii* strains to such substances is currently limited. To our knowledge, no data are available on minimal inhibitory concentrations or resistance mechanisms related to benzene derivatives in these strains. However, environmental isolates of *A. baumannii* have been reported to utilise aromatic compounds such as benzene as a carbon source, indicating potential metabolic adaptability under specific conditions (63).

**Figure 4:**
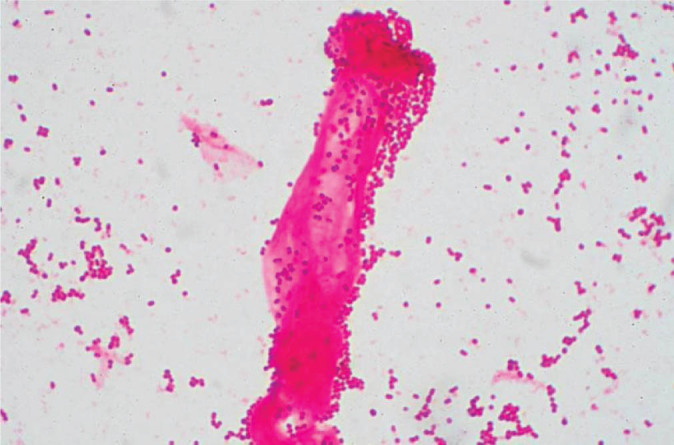
Immobilised cells of *A. baumannii* Sava 4 on the surface of microplastic particle at 1000× magnification

**Figure 5:**
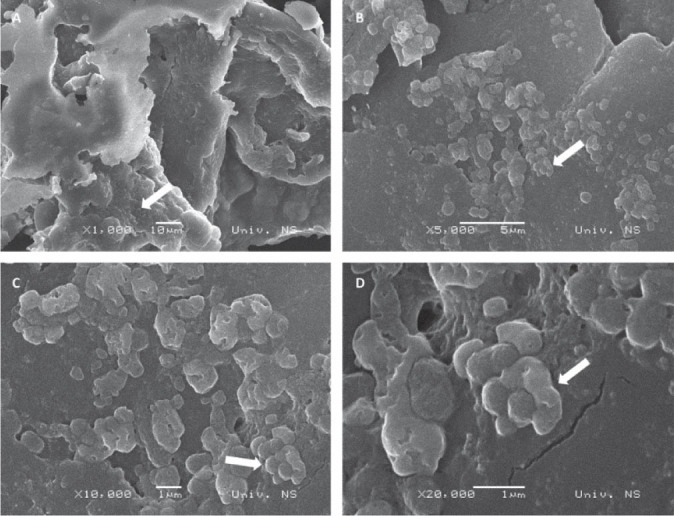
SEM images of PE microplastic particles after incubation with *A. baumannii* Sava 4 at increasing magnifications illustrating the surface morphology of the particles and the distribution of bacterial cells on microplastic surface

Furthermore, PE microparticles seem to favour the immobilisation of *A. baumannii* Sava 4 and the formation of biofilms, as shown by microplastic surface microscopy (Figures 4 and 5). These results are consistent with reports of pathogens such as *Vibrio* spp. (64), *Pseudomonas* spp. (65), and *Acinetobacter* spp. (66) on microplastics. However, the bacterial community on microplastics has mainly been studied using culture-independent methods, which provide limited information on antibiotic resistance phenotypes and virulence potential (26). Therefore, future studies should focus on culturing “plastisphere” microorganisms and characterising their properties. PE microplastics can serve as a substrate for bacterial attachment and biofilm formation even without adsorbed pollutants, as shown in previous studies (67, 68).

Note, however, that microscopy could not definitively confirm biofilm formation or differentiate between live and dead cells on microplastic surfaces. Instead, the observed structures should be interpreted as indicative of bacterial attachment and possible biofilm-like organisation. Besides, sample preparation for optical microscopy did not include washing steps to remove non-attached cells, which may have affected the interpretation of bacterial distribution on particles. Still, the proliferation of *A. baumannii* Sava 4 on microplastics was confirmed by cultivation-based methods, and the successful colonisation of this pathogen poses the risk of carbapenemase genes that confer resistance to other bacteria (68, 69). It has been shown that *A. baumannii* Sava 4 can survive unfavourable environmental conditions, including fluctuations in temperature, pH, oxygen, and nutrients for extended periods (45, 46), and our study has also evidenced that it can resist benzene derivatives. Future studies should expand research to a wider array of inorganic and organic pollutants, types of microplastic, and pathogens. In addition, real-life samples, such as those from wastewater, should be included to capture a more realistic environmental dynamic. The case in point is the use of the Mueller-Hinton broth in our study, which ensures reproducible conditions and sufficient biomass for reliable quantification but may have enhanced bacterial resilience and reduced the effects of contaminants adsorbed on PE microparticles. In contrast, oligotrophic media, such as mineral salt medium, may better reflect environmental conditions that typically do not support significant proliferation of heterotrophic bacteria such as *A. baumannii*.

Besides the limitations of the controlled environment discussed above, our study involved only one bacterium isolate, which excludes real-life competition on PE surfaces, and only one type of polymer, both of which preclude generalisation to other bacterial strains, polymer types, or environmental contexts.

## CONCLUSION

Despite these limitations, our study has clearly shown that microplastics may play a key role as vectors for the transmission of pathogens in aquatic environments and that *A. baumannii* Sava 4 proliferation and biofilm formation on PE microparticles is not affected by the presence of benzene derivatives. Microplastics, in fact, pose a dual environmental risk – as carriers of organic pollutants and antibiotic-resistant pathogens.

Further research is needed to map the transmission of different pathogen species to aquatic food sources and their potential long-term effects on human health.
